# An electronic patient-reported outcome created based on my needs is worth using: an explorative qualitative study investigating young people’s opinions for a health assessment tool

**DOI:** 10.1186/s41687-022-00436-z

**Published:** 2022-03-28

**Authors:** Petra V. Lostelius, Magdalena Mattebo, Anne Söderlund, Åsa Revenäs, Eva Thors Adolfsson

**Affiliations:** 1 Clinic for Pain Rehabilitation Västmanland, Region Västmanland, Västerås, Sweden; 2 School of Health, Care and Social Welfare, Mälardalen University, Västerås, Sweden; 3 Centre for Clinical Research Region, Hospital of Västmanland Västerås, Region Västmanland – Uppsala University, 721 89 Västerås, Sweden; 4 Orthopedic Clinic, Region Västmanland, Västerås, Sweden

**Keywords:** Young people, Medical informatics, Electronic patient-reported outcome, Youth health clinic, Qualitative research

## Abstract

**Background:**

Young people in different healthcare settings are positive about using electronic patient-reported outcomes (ePROs), which are meant to increase the effectiveness and safety of interventions from the patient’s perspective. Sweden offers free healthcare to young people aged 12–25 years at 275 youth health clinics (YHCs), whose goals are to strengthen young people and promote sexual, physical, and mental health. YHCs need effective ways to identify the overall picture of young people’s health and health-related problems. To our knowledge, there is no ePRO for YHCs that provides an overview of young people’s health from several health perspectives. The aim of this study was to explore young people’s view on content and design of an ePRO to provide an overview of their health and health related problems when visiting a YHC, and their opinion on what healthcare needs to consider when using the ePRO. This was an explorative qualitative study. The participants were included from five YHCs, in different socioeconomic areas in central Sweden. Fifteen participants were included: 10 girls, three boys, and two non-binary participants with an age range of 16–22 years. Data were collected using a semi-structured interview guide and individual interviews, and inductive content analysis was performed.

**Results:**

One main theme, “ePRO created based on my needs is worth using” and two sub-themes, “Appealing content and design” and “Trusting healthcare”, emerged. The participants wanted that an ePRO should include overall questions about mental-, physical-, and sexual health and social support. Participants also believed the ePRO must disclose the risks of self-harm or suicide. The participants noted the importance of emotional and digital security when using the ePRO and having a confidential conversation with a healthcare provider. To share health information means to trust to gain health.

**Conclusions:**

The study participants' views on content and design can form the basis for designing an ePRO for young people. Their thoughts on safety and treatment in healthcare can be considered in the development process. This study is the starting point for developing an ePRO for young people at YHCs.

## Background

The Public Health Agency of Sweden reports that most adolescents and young adults (young people) in Sweden have good health, but mental health problems and psychosomatic symptoms are increasing [1]. The years of adolescence and young adulthood are fundamental for personal development, future workforce productivity, and decision making [2–4], and it is therefore important to identify poor health in young people at an early age.

The patient-reported outcome (PRO) measure collects self-reported health data and functional status directly from patients, without interpretation by the clinician [5]. PROs are used in clinical practice to assess symptoms, functioning, and well-being in a systematic way [5, 6] and to promote patient-centered care, facilitate communication within multi-disciplinary teams and between the patient and healthcare providers, aid decision-making, and monitor patient care [6, 7]. The electronic development of the PRO, called the electronic patient-reported outcome (ePRO), is convenient for patients by allowing them the freedom to choose the location and time to answer questions. ePROs inform healthcare providers in advance of a patient/client meeting, have been shown to be feasible in different healthcare settings and increase the effectiveness and safety of interventions [8, 9]. Using an ePRO to identify psychosocial problems can increase young peoples’ reporting of health-related problems, make it easier to share their feelings, despite being vulnerable [10], and has shown good results in terms of administration time and detection rates [11].

Repeated measurements with PROs that are followed by feedback from healthcare providers can help to accelerate the treatment effects [12]. Young people have stated that using a PRO before a healthcare appointment allowed them more time to talk about what is most important to them during the appointment [10]. Examples of PROs for young people that are feasible, accepted, and used clinically are the SEXual health Identification Tool (SEXIT), developed to identify Swedish young people exposed to or at risk of poor sexual health [13, 14], and YouthCHAT, with purpose to identify psychosocial and mental health issues in young people in New Zealand [11, 15, 16]. As PROs/ePROs are increasingly recognized for their utility, platforms of PROs/ePROs are being created, for example the Patient-Reported Outcome Measurement Information System® (PROMIS®) [7].

Sweden offers free healthcare to young people aged 12–25 years at about 275 youth health clinics (YHCs). YHCs are multi-disciplinary clinics that are staffed with midwives, counselors, and a doctor [17] and sometimes additional members, such as nurses, dieticians, and psychotherapists. All YHC activities are governed by the Social Services Act (SFS 2001: 453) [18] and the Health Care Act (SFS 2017: 30) [19]. The YHC’s goal is to strengthen young people and promote sexual, physical, and mental health [20]. The focus of the health assessment at the YHC depends on the reason for the appointment and local clinical decisions at the YHC and different PROs are used to collect information about young people’s health [20]. To optimize their work effort and increase equity in treating young people, YHCs need to develop new and structured ways to identify health-related problems [21]. A tailored-designed tool can potentially contribute to behavioral change, improved health, and increased self-efficacy [22] for self-care actions.

To our knowledge, there is little understanding of young people’s opinions about the use of ePROs at YHCs and what should be included. Thus, the aim of this study was to explore young people’s view on content and design of an ePRO to provide an overview of their health and health-related problems when visiting a YHC, and their opinion on what healthcare needs to consider when using the ePRO.

## Methods

### Study design

This study used an exploratory design and inductive qualitative content analysis of Graneheim and Lundman [23] to gain new knowledge from individual interviews. The study was approved by the Regional Ethics Committee, Uppsala, Sweden and was performed according to the principles of the Declaration of Helsinki [24].

### Participants and setting

Young people participated from five YHCs. The participants had different socioeconomic status and were purposively recruited from two regions in central Sweden. Heterogeneity was desired regarding gender, age, socioeconomic status, cultural background, and sexual orientation. The inclusion criteria were the ability to speak Swedish, aged 16–23 years, and having visited a study YHC.

YHC healthcare providers asked young people to participate in the study when meeting for an appointment. If interested, potential participants were informed about the study verbally and in writing and were asked for permission for the first author to contact them to set up time for an interview.

### Data collection

The study was conducted from June through November 2018, when saturation in data collection was reached. The participants gave their informed written consent to participate before the individual interviews started. A semi-structured interview guide was used for the interviews (Table 1). The interviews were conducted by the first author (who had extensive experience from clinical healthcare but no relationship with any participant). The interviews focused on exploring what was important in an intended ePRO for use at the YHC, identifying health and healthrelated problems. The participants’ thoughts linked to the planned topic and questions were explored. The interviews ranged in length from 30 to 60 min. All interviews were audio-recorded and transcribed verbatim.

**Table 1:** Interview guide topics and questions used to investigate participants’ views on electronic patient-reported outcome (ePRO) at youth health clinics (YHCs)

Topics	Questions	Sub-questions
ePRO content and design	What health areas are important to identify to give a picture of young people’s health?	What types of questions should NOT be included in an ePRO?
		What type of questions SHOULD be part of an ePRO?
		How long is okay to spend on answering ePRO questions?
Participants’ suggestions for healthcare	What is important for healthcare services to best find out about young people’s health?	What is needed/important to meet the needs of young people and to support healthy behaviors?
		What improvements can be made for the YHC to best care for young people?
ePRO pros and cons	Could you tell me about the pros or cons of using an ePRO at the YHC?	How would it be negative for an ePRO to convey your health status?
		How would it be positive for an ePRO to convey your health status?

### Data analysis

Inductive content analysis was used to interpret the meaning of the interviews and to develop an understanding [25] of what would be important for the participants when using an ePRO at the YHC [23]. The transcribed interviews were read several times to gain familiarity with the data. Initially, two members (PVL, ÅR) of the research team read three transcripts separately, identified meaning units, and transferred them into codes. The codes were compared between the two researchers to strengthen the credibility. The first author continued to code the remaining twelve interview transcripts and printed the codes on paper notes. Then, all researchers collectively sorted the codes into preliminary categories (what the text said) based on the content and with the focus on the study aim. The first author proceeded with the analysis in further categorizing the codes, while staying in continuous dialogue with the other researchers to ensure that the data was correctly labelled and sorted [23]. The abstraction process led to the development of sub-themes and a theme by describing the latent content in the data (i.e., what the text talked about). Examples of the schematic analysis process are given in Table 2.

**Table 2:** Examples of the schematic analysis process

Meaning unit	Condensation	Code	Sub-category	Category	Sub-theme	Theme
If they just say that I should use digital support, then I must first know the purpose, like, well because, it’s like you’re telling me	It’s important to know the purpose of ePRO	Important to know the purpose	To understand why	Content based on young people’s needs	Appealing content and design	An ePRO created based on my needs is worth using
They can get a larger picture and, maybe, those who take care of it, can see the most important things and put extra focus on them	See the large picture and what is most important and focus on that	To be able to focus on what is important	The meeting will concern what is important	ePRO supporting the face-to-face meeting	Trusting healthcare	

*ePRO* electronic patient-reported outcome

## Results

A total of 15 young people participated in the study. The participants’ demographics are detailed in Table 3.

**Table 3:** Participants’ demographic information

IP	Age	Housing	Lives with	Birthplace	Parents’ birthplaces	Gender	Sexual orientation	Education
1	17	Villa	M, F, S	Sweden	Sweden	Girl	Heterosexual	High school
2	22	Apartment	F, S	Sweden	Sweden and Nordic Country	Nonbinary	Bisexual	High school
3	17	House	M, F	Sweden	Sweden	Girl	Homosexual	High school
4	17	House	M, F, S	Sweden	Sweden	Girl	Heterosexual	High school
5	21	Student apartment	Alone	Sweden	Sweden	Girl	Heterosexual	University
6	18	Villa	M, F, S or partner	Sweden	Sweden	Girl	Heterosexual	High school
7	18	Villa	S or other adult	Sweden	Sweden, Outside E	Girl	Heterosexual	High school
8	19	Villa	M, F	Sweden	Sweden	Girl	Heterosexual	High school
9	22	Rental apartment	Alone	Sweden	Sweden	Boy	Bisexual	University
10	19	Villa	F	Sweden	Sweden	Girl	Heterosexual	High school
11	18	Villa	M, F, S	Sweden	Sweden, OEC	Girl	Heterosexual	High school
12	22	Student apartment	Alone	Sweden	Outside E	Boy	Heterosexual	University
13	21	Student apartment	Alone	Sweden	Sweden	Girl	Heterosexual	University
14	19	Townhouse	M, S	Sweden	Outside E	Boy	Heterosexual	Adult high school
15	17	Villa	M, F, S	Outside E	Sweden	Girl	Heterosexual	High school

*IP* interview person, *M* mother, *F* father, *S* sibling, *OEC* other European country, *E* Europe

**Fig. 1:**
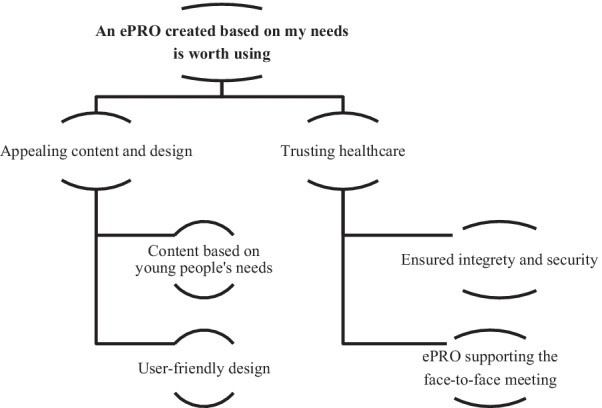
Overview of the result, presenting the theme at the top and the two sub-themes with two categories each below

One main theme “An ePRO created based on my needs is worth using” and two sub-themes “Appealing content and design” and “Trusting healthcare” (Fig. 1), emerged in the data analysis that explored young people’s view on content and design of an ePRO to provide an overview of their health and health-related problems when visiting a YHC, and their opinion on what healthcare needs to consider when using the ePRO. The two sub-themes with included categories are presented in the text below with participants’ views illustrated with citations.

### Sub-theme ‘Appealing content and design’ and two categories

The participants stated that an ePRO designed and developed with and for young people could have the potential to give an accurate picture of young people’s health status. They wanted the ePRO to provide an overview of their mental-, physical-, and sexual health, as well as social support. However, they also wanted the ePRO to identify risks of self-harm and suicide. The ePRO design should be non-judgmental, easygoing and undemanding to ensure that young people would find it interesting enough to use. The participants pointed out that the ePRO should be inclusive and not force young people into fixed categories based on, for example gender or sexual orientation.

#### Content based on young people’s needs

The participants believed that a known and clear purpose of answering the ePRO would increase their willingness to report their health status. The ePRO should be investigative, curious, and provide an overview of young people’s health by addressing mental-, physical- and sexual health topics. The participants also found it important to include questions about social support.

Kind of simple, to be able to get, well, a good overview at least. (IP13)

Although the participants thought that the ePRO should generally ask broad health questions, some of the participants thought that it should also identify some factors in depth but also to delve further with specific questions. For instance, the ePRO mental health area should include questions about self-harm and suicide. However, all participants were positive towards the health information that the ePRO had potential to produce.

So, I think you have to ask about it [self-harm, eating disorders, and suicide] … Where else should you ask about it? It is not possible to… forget… that it actually exists. So yes, I think you should ask such questions, I think they are the most important. (IP8)

#### User-friendly design

The participants agreed that it must be easy to understand and use the ePRO. They wanted no mandatory questions and had several suggestions on how to best formulate the responses; some wanted yes-or-no questions, whereas others wanted open-answer questions and the freedom to choose how to respond to the questions in the ePRO.

For some, it is enough [with yes or no], and some want to write, so mixed responses, and both open and with one answer. Because it’s different from person to person. (IP4)

The participants thought that too straightforward questions could cause shame or pressure and make young people hide their true feelings or decline using the ePRO. There were also concerns if some health questions could make young people feel uneasy, and a skepticism that the ePRO would be able to reflect their varying day-to-day health status. Furthermore, they warned that a boring ePRO design could make young people lose interest and stop answering, and that the ePRO would fail to capture young people’s health status. Using pictures and keeping the response time to no longer than 10 min were examples of ways to keep their interest.

… after all, many people do not like a lot of text - like me… if I read a long text, I just get tired of it, so if a picture comes in at the beginning, it would be more fun… (IP10)

In addition, the ePRO needed to be nonjudgmental and to make no assumptions about gender, sexual orientation, number of sex partners, or girlfriends or boyfriends. Instead, questions should be open and inclusive.

### Subtheme ‘Trusting healthcare” and two categories

The participants recognized that answering questions about their health could make them vulnerable in different ways. They expressed a need for personal contact with the healthcare providers, ensuring support, empathy, and confidentiality. The participants stated that meaningful ePRO health questions may empower and validate young people and increase their self-knowledge. Hence, the participants also realized that the ePRO could give an opportunity for young people to be more open and share their feelings with the YHC’s healthcare providers, which would lead to better communication between them.

#### Ensured integrity and security

The participants thought that answering an ePRO in the waiting room could make young people feel exposed. Some of the participants wanted to answer the ePRO questions at home, and others suggested using a separate area in the waiting room or a consulting room at the clinic.

So… I know that there is privacy… but preferably it should say somewhere that… “This is just between us working here and you.” I think many would feel safer and want to participate. (IP14)

The participants declared themselves as uncomfortable with the potential risk of someone hacking the information in the ePRO or online. A few participants also mistrusted healthcare providers to maintain confidentiality, during lunch of coffee breaks. They also were concerned that answering the ePRO questions might lead to personal disadvantage, for instance, that only those considered to be worst off would be offered support.

You don’t base it on yourself, but instead on others, like “This person is worse off than I am because he/she has a self-harming behavior and I don’t.” (IP13)

#### ePRO supporting the face-to-face meeting

It was important to the participants that the ePRO should not replace the face-to-face meetings. The participants expressed a need to know that their problems would be received and taken seriously. They confessed that they wanted validation and empathy from healthcare providers to help them feel safe when sharing their feelings and thoughts.

…for me personally, I think it’s easiest to talk to someone I feel safe with… Someone who seems to care, like a little bit… like, a confident person who is kind and nice and listens. I think that’s important. (IP7)

The participants expressed that using the ePRO for health status reporting could make it more difficult for young people to hide from their problems. The participants thought that answering health questions could prepare them better for meeting with the YHC healthcare providers. Hence, the ePRO would provide time for self-reflection, which would lead to greater self-knowledge and self-awareness. This was considered as positive.

To, like, see my answers and get an overview may provide the hindsight of… “oh, is this how I really feel.” And like… “maybe I should ask to talk to someone.” (IP3)

The participants liked that the ePRO could inform healthcare providers about a young person’s health, in advance of the consultation. They believed that it would help overcome the challenge some young people may have of talking about themselves. The ePRO could potentially take the pressure off them to verbalize their problems and concerns and automatically transfer the initiative to address relevant health issues from the young person to the healthcare provider. They were positive that ePRO would direct healthcare providers toward the most important health issues.

It would be positive for me to not have to spend several hours just talking about my history in some way and get started quickly to work on the problems that are actually more… in the present… That way I think it helps quite a lot. (IP2)

## Discussion

This study’s main finding was the theme “An ePRO created based on my needs is worth using”. This entails that young people want healthcare to target their efforts towards young people’s needs and create an ePRO that young people want to use. For this, the participants stated that the ePRO should be appealing to themselves and give healthcare providers a general picture of young people’s health, as well as deeper knowledge about their mental health. The participants were positive toward the ePRO potential to transfer some of the responsibility to initialize a conversation about what is hardest to talk about. Ultimately, the participants believed that meaningful ePRO health questions may lead to benefits for young people.

The sub-theme, “Appealing content and design” relates to what was considered important about the health questions of the ePRO. The participants noted the importance of the ePRO giving an overview of their health within four areas; mental-, physical -, sexual health and social support. However, the portraying of the “the big health picture” did not minimize the focus on severe mental health issues and lifted out the importance of asking about the risk for self-harm and suicide in young people. These findings are congruent with previous research, claiming that young people think that ePROs can provide more time to focus on what’s really important to them [10]. The importance of focusing on mental health at YHCs is supported by Goicolea et al. [21], who found that YHCs may play an important role in promoting young people’s mental health. It has been reported that asking about suicide can reduce the risk of suicide [26], and the increasing number of young people with a need for inpatient healthcare [1] makes it an essential topic to address. Vulnerable groups such as sexually active homosexual or bisexual young people have a higher risk of suicide thoughts or attempts [27], which points to the need for appropriate mental health information at YHCs. The participants had also opinions of what questions and response options that were optimal. Some participants wanted the ePRO to contain open-ended questions. Although research suggests that open-ended questions can add dimensions to standardized ePROs [28], when developing a new ePRO, the details provided by open-ended questions must be balanced by the extra time needed to answer and interpret the answers.

The sub-theme “Trusting healthcare” includes the participants’ hesitation and hopefulness of using an ePRO. They were doubtful to take the risk of being exposed to software hackers, which exposes the participants’ lack of trust in digital security. These concerns have also been recognized in a multinational study of young people using ePROs for health status reporting [29]. The participants were apprehensive of being observed by peers in the waiting room and were also suspicious of trusting the healthcare providers to maintain professional confidentiality. This lack of security for medical confidentiality can represent a threat to young people’s health if they withhold important information about themselves. The finding gains strength from a scientific review that noted that patients’ understanding of and beliefs about medical confidentiality are often incongruous with the writings of practitioners or legal experts [30]. Although the participants felt uncomfortable sharing their inner concerns and emotions, they were confident that meeting face-to-face with healthcare providers was important, which is supported by previous research [10, 15]. Previous research has found that receiving reports before a healthcare visit could help healthcare providers to initiate a conversation about psychosocial issues [15] and promote communication and co-decisions [6, 7] with young people. Furthermore, it has been reported that young people are positive towards healthcare providers asking questions about things that may be difficult to talk about, for example, negative experiences of sexuality and sexual risk-taking [13, 31–33] and the possibility for shared planning and decision-making [15]. The SEXIT tool is an example of an evidence-based tool that is already used at many YHCs. SEXIT has been found feasible and acceptable by YHC healthcare providers, and encourages communication about sexual health between young people and healthcare providers [13, 14]. This study’s participants said that young people would need to know the reason to use the ePRO and had at the same time hopes that the ePRO would entail benefits making it worth to use. These findings are supported by research concluding that the reason for and the value of using PROs are important to young people [29].

Patient involvement in healthcare development is not self-evident [34] but needs to be considered as a right, in line with the United Nations’ Convention of the Rights of the Child [35]. The engagement of the young participants in this study was the starting point of a developmental process to produce an ePRO for use at YHCs in Sweden to identify health and health-related problems in young people who visit YHCs. YHCs work holistically and are person-centered and strive to use evidence-based measures and treatments [20]. The participants in this study wanted the YHC health assessment to include a wide perspective of health and include mental-, physical-, sexual health and social support.

The study was conducted based on Swedish YHC conditions, which includes both adolescents and young adults. One limitation of the study was that the youngest YHC visitors were not represented because of ethical considerations. Another study limitation was that no records were kept of the young people who declined to participate because of the clinical conditions at the YHCs. The findings in this study, add to the research body by indicating the benefits of implementing ePROs at YHCs in Sweden.

Despite of limitations, the overall data are reflective of the YHC patient population, and the 15 included interviews were rich of information and no new perspectives emerged in the final interviews. Qualitative content analysis, as described by Graneheim and Lundman [23], was chosen to give structure to the text and analysis. The quotations contribute to the reader’s opportunity to judge the study’s trustworthiness. All considered, the study holds good credibility throughout the methodology [36] from the aim through the analysis. Overall, the existing participant population makes the results transferrable to all Swedish YHC settings.

## Conclusions

Young people have valuable opinions about the use and content of an ePRO for YHCs. The participants in this study wanted an ePRO to provide an overview of their health and guide healthcare providers to address serious mental health issues. Understanding why they should use an ePRO and that the information in the ePRO would form the basis of the conversation at the YHC, was more important than the potential inconvenience for young people. The findings highlight the importance of including young people when developing an ePRO for use in YHCs and the focus on the user’s perspectives. These results will serve as the starting point for the development of an ePRO for Swedish YHCs.

## Data Availability

Deidentified data may be available from the corresponding author upon request. The study is committed to the General Data Protection Regulation (GDPR) and the Swedish Ethical Review Authority.

## Abbreviations

ePRO: Electronic patient-reported outcome
PRO: Patient-reported outcome
SEXIT: SEXual health Identification Tool
YHC: Youth health clinic
